# Manganese (Mn) doping effects on the structure and surface characteristics of copper zinc tin sulphide (CZTS) transition metal sulphides synthesised *via* a sol–gel method

**DOI:** 10.1039/d5ra09688j

**Published:** 2026-03-27

**Authors:** Ahamed Razeek Najitha, Siti Rudhziah Che Balian, Hassan Ahmoum, Fatin Saiha Omar, Puvaneswaran Chelvanathan, Mohd Sukor Su'ait

**Affiliations:** a Department of Physical Sciences, Faculty of Applied Sciences, South Eastern University of Sri Lanka (SEUSL) 32200 Sammanthurai Sri Lanka najithaaseeque@seu.ac.lk; b Solar Energy Research Institute (SERI), Universiti Kebangsaan Malaysia (UKM) 43600 Bangi Selangor Malaysia mohdsukor@ukm.edu.my; c Centre of Foundation Studies, Universiti Teknologi MARA Cawangan Selangor, Kampus Dengkil 43800 Dengkil Selangor Malaysia; d Faculty of Medicine and Pharmacy, Ibn Zohr University BP 32/S Agadir 80000 Morocco; e Department of Applied Physics, Faculty of Science and Technology, Universiti Kebangsaan Malaysia (UKM) 43600 Bangi Selangor Malaysia; f Battery Technology Research Group (UKMBATT), Faculty of Science and Technology, Universiti Kebangsaan Malaysia (UKM) 43600 Bangi Selangor Malaysia

## Abstract

Transition metal sulphides have emerged as promising candidates for a range of electronic and energy-related applications owing to their tunable characteristics. This study investigates the effects of manganese (Mn)-doped copper zinc tin sulphide (Cu_2_ZnSnS_4_, CZTS) on its structural and surface properties. Mn-doped CZTS samples were synthesised *via* a sol–gel method at various doping percentages. The resulting samples were sulphurised prior to the physicochemical analyses. X-ray diffraction (XRD) confirmed the formation of a tetragonal phase, with noticeable lattice expansion due to Mn incorporation. The crystallite size increased from 24.52 nm to 42.01 nm at low Mn doping levels, while the degree of crystallinity decreased to 50–65%. Correspondingly, the strain and dislocation density were reduced to 3.32 × 10^−3^ rad and 0.57 × 10^−3^ (nm)^−2^, respectively. Raman analysis verified a stannite-dominant structure, with red shifts corroborating the XRD findings. Although the surface areas and pore volume of CZTS samples decreased upon Mn doping, the pore size showed a notable increase. In addition, the particle size distribution ranged between 1 and 2 µm, and the zeta potential shifted from a net negative to a net positive charge, thereby enhancing the charge mobility after Mn doping. Scanning electron micrographs revealed reduced particle agglomeration, improved grain size, and surface uniformity following Mn doping, while energy-dispersive X-ray analysis confirmed the successful Mn substitution into the CZTS in all samples. In conclusion, Mn doping effectively modified the lattice structure and surface characteristics of CZTS, indicating its potential to improve the material's performance in electronic devices, energy storage, and energy harvesting applications.

## Abstract 2

Doping of larger Mn ions at Zn sites in CZTS induces lattice expansion and modifies structural parameters, leading to improved performance in energy-related applications.

## Graphical abstract

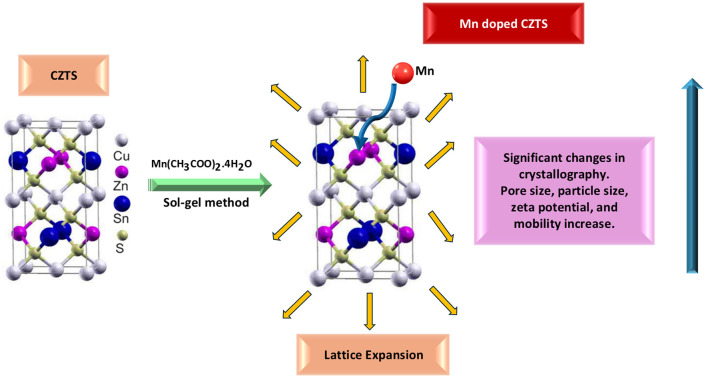

## 1. Introduction

Copper zinc tin sulphide Cu_2_ZnSnS_4_ (CZTS) is a transition metal chalcogenide that has emerged as a highly promising material due to its elemental abundance, environmental safety, and lower toxicity compared to many other metal chalcogenides.^1–4^ Copper indium gallium selenide (CIGS) and cadmium telluride (CdTe) are two common photovoltaic chalcogenides that rely on either rare and expensive indium and gallium or toxic cadmium.^5^ In contrast, CZTS is composed entirely of naturally abundant elements, namely copper (Cu), zinc (Zn), tin (Sn), and sulphur (S). Other commonly used chalcogenides, including CdS, CdSe, and PbS are also toxic, further highlighting the environmental advantages and safety concerns of CZTS.^6^

CZTS material has been employed in a wide range of applications, including photovoltaics,^7–9^ catalysis,^10^ sensing,^11^ optoelectronics,^12^ energy storage,^13–16^ thermoelectric,^17^ and water splitting.^18^ Several methods have been proposed to improve the efficiency and performance of CZTS material, such as alkali-doping,^19,20^ cation substitutions,^21^ and the incorporation of polymer composites and carbon-based material.^13^ The use of transition metals as dopants alters intrinsic optoelectronic and electrochemical properties of CZTS.^22^ Compared to other transition metals such as nickel (Ni), cobalt (Co), and copper (Cu), manganese (Mn) is a more suitable material to replace Zn because the ionic radius of Mn^2+^ (0.8 Å) is higher than Cu^+^ (0.74 Å), Co ^2+^ (0.72 Å) and Ni^2+^ (0.69 Å).^23^ El Mahboub *et al.*^24^ reported that Co-doped CZTS exhibited XRD peaks to higher diffraction angles, corresponding to reduction in the crystal lattice due to the substitution of Zn^2+^ (0.74 Å) with smaller Co^2+^. They observed decreased lattice parameters, a phase transition from kesterite to stannite, and a reduction in point defects at low doping levels, followed by an increase at higher concentrations.^24^ A previous study by Digraskar *et al.*^25^ investigated Ni-doping CZTS and similarly reported XRD peak shifts attributed to decreased lattice parameters. It is well established that introducing dopant ions changes the interatomic distances in the host material. Dopants with different ionic radii modify lattice parameters and crystal structure, thereby affecting the electronic properties of the material.^25^ The substitution of large Mn ion into Zn is expected to expand the CZTS lattice and modify its properties. Furthermore, Mn possesses a more stable electron configuration than other transition metals. The electron configuration of Mn, Ni, and Co are 4s^2^ 3d^5^, 4s^2^ 3d^8^, and 4s^2^ 3d^7^, respectively. The Mn^2+^ oxidation state is particularly stable due to its half-filled 3d^5^ orbitals. According to the Pauli exclusion principles,^26^ the five valence electrons in Mn occupy the five d orbitals with parallel spins, conferring intrinsic stability of Ni or Co.^27^ Additionally, Mn exhibits multiple oxidation states and favorable catalytic behavior, making it attractive for electrochemical energy storage applications.^28,29^

Several studies have examined Mn doping in transition metal chalcogenides. Lie *et al.*^27^ investigated Mn-substituted Cu_2_Mn_*x*_Zn_1−*x*_Sn(S,Se)_4_ for solar cell applications.^27^ Mn incorporation into the lattice provides positive improvement, and this was confirmed by shifts in XRD and Raman peaks. The optimal composition was found at *x* = 0.05. Cui *et al.*^30^ studied Mn-doped Cu_2_ZnSn(S,Se)_4_.^30^ The XRD analysis revealed that even though Mn^2+^ ions did not integrate into the lattice and induced impurity phases, the intensity of XRD peaks increased, indicating improved crystallinity. Moreover, Mn doping reduced bulk resistance and enhanced the electrical properties of CZTSSe, with an optimal Mn doping ratio of 0.03. Additional trace amount of Mn further enhanced the carrier transport within the films and increased surface current.^30^ Shinde *et al.*^31^ reported the electrical and structural behaviour of Mn-doped CoS synthesised using the SILAR method. XRD analysis showed that peak positions remained unchanged after Mn doping, although the peak intensity increased. Films doped with 3% Mn exhibited higher crystallinity and potentially better electronic conductivity compared to pure CoS. Mn incorporation enhanced the surface area, reactive surface sites, areal active surfaces, and current density of the films.^31^

Mote *et al.*^32^ investigated the Mn-doped ZnO materials and observed that lattice parameters and unit cell volume increased linearly with Mn concentration, while the XRD peaks shifted slightly due to doping. The enhancement in lattice constants was attributed to lattice strain arising from the ionic radius between Zn^2+^and Mn^2+^.^32^ Raba-Páez *et al.*^33^ explored tungsten (W)-doped CuO synthesised *via* co-precipitation. The XRD peaks shifted slightly towards lower angles due to tensile stress, and peaks broadening indicated smaller particles formation, as confirmed by FESEM and TEM analyses. Although lattice parameters ‘*a*’ decreased and ‘*b*’ increased with increasing tungsten content, the unit cell volume remained relatively unchanged. BET measurements revealed increased specific surface area and pore size, while DLS analysis indicated reduced particle size after doping.^33^ Collectively, these studies demonstrate that doping significantly influences the structural, morphological, and surface properties of materials, thereby altering their physicochemical behaviour.

Although various studies have shown that Mn doping can effectively modify the structural, electronic and electrical properties of numerous chalcogenides and transition metal oxides, a systematic investigation of Mn incorporation into CZTS by sol–gel method, with comprehensive structural, morphological and physicochemical analysis, remains limited. Existing studies predominantly focus on thin-films systems or provide only partial structural evaluations. A thorough crystallographic analysis combined with systematic assessment of surface area, particle size, and electrical properties has not been extensively reported. The literature lacks a comprehensive and quantitative evaluation of Mn doping effects on the structural, morphological, and physicochemical properties of CZTS in powder form. Therefore, a systematic investigation is essential to understand how Mn incorporation changes the lattice structure, microstructure, surface properties and physicochemical properties of CZTS. Addressing these gaps will provide valuable insights into advancing Mn-doped CZTS as a versatile, high-performance material for energy related applications.

The aim of this work is to investigate the influence of manganese (Mn)-doped Cu_2_(Zn_1−*x*_Mn_*x*_)SnS_4_ at various percentages ratios *via* sol–gel method on its physicochemical properties through structural, morphological, compositional and surface analyses. Doping Cu_2_ZnSnS_4_ (CZTS) with manganese ions, which have a larger ionic radius than zinc ions, is expected to expand the crystal lattice and moderately influence defect formation. This substitution may modify crystallinity, promote more uniform particle distribution, and improve charge transport properties (conductivity, diffusion, and mobility). Consequently, manganese incorporation is expected to enhance the overall structural integrity and physicochemical performance of the CZTS.

## 2. Experimental

### 2.1. Materials

Chemicals used for the Mn doped CZTS synthesis include copper(ii) acetate monohydrate, (Cu(CH_3_COO)_2_·H_2_O, 98%, Nacalai Tesque Inc., Japan), zinc(ii) acetate dihydrate (Zn(CH_3_COO)_2_·2H_2_O, 98%, Nacalai Tesque Inc., Japan), manganese(ii) acetate tetrahydrate (Mn(CH_3_COO)_2_·4H_2_O, 99%, Sigma-Aldrich, USA), tin(ii) chloride dihydrate (SnCl_2_·2H_2_O, 95%, Nacalai Tesque Inc., Japan), thiourea (CH_4_N_2_S, Nacalai Tesque Inc., Japan), 2-methoxyethanol (99.8%, Sigma-Aldrich, US), monoethanolamine (MEA) (97.0%, Nacalai Tesque Inc., Japan), and colloidal sulphur (Nacalai Tesque Inc., Japan).

### 2.2. Synthesis of Mn doped CZTS

**Fig. 1:**
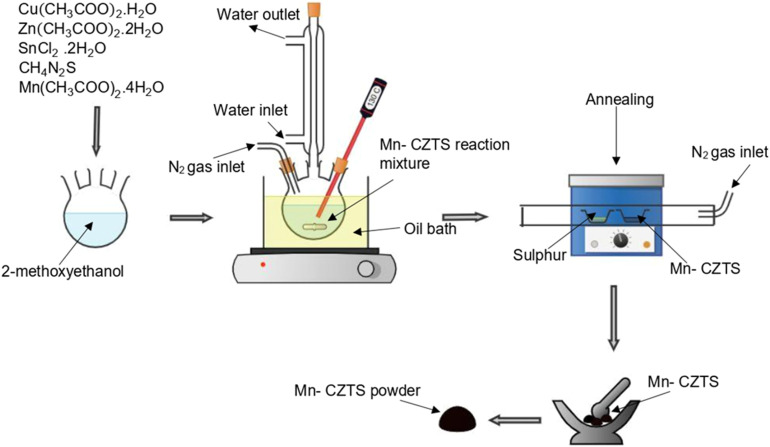
Synthesis of Mn-doped CZTS powder.

Sol–gel technique was used for the synthesis of manganese (Mn)-doped Cu_2_(Zn_1−*x*_Mn_*x*_)SnS_4_ at various concentrations of Mn (*x* = 0, 0.03, 0.1, 0.17, 0.3, 0.5, and 1.0) as reported by Ahmoum *et al.*^34^ and Hassan *et al.*,^14^ with slight modifications to the synthesis conditions. A solution containing 1.1 M of Cu(CH_3_COO)_2_·H_2_O, 0.7 M of Zn(CH_3_COO)_2_·2H_2_O, 0.6 M of SnCl_2_·2H_2_O and 2.4 M thiourea was prepared in a three-neck round-bottom flask.^35^ 2-Methoxyethanol was used as a solvent, and the required Mn precursor was added to the desired doping concentration. Monoethanolamine (MEA) was used as a basic media and stabiliser.^36^ The resulting solution was heated at 130 °C for 2 hours in an oil bath to facilitate the sol–gel transformation. The obtained slurry was subsequently transferred to a tubular furnace and annealed at 550 °C for 3 hours under a sulphur atmosphere with continuous nitrogen.^37–40^ Finally, the Mn-doped CZTS product was crushed using a mortar and pestle to obtain a fine powder. The synthesis process of Mn-doped CZTS powder is illustrated in Fig. 1.

### 2.3. Physicochemical characterisation

Several characterisation techniques were employed to analyse the structure, morphology, and properties of CZTS. The X-ray diffraction (XRD) analysis was performed using a Bruker D8-Advance diffractometer using Cu Kα as the X-ray source (*λ* = 1.5406 Å). Crystallographic parameters, including interplanar spacing (*d*), lattice position (*a*, *c*), volume (*V*), strain (*ε*), dislocation density (*δ*), full width at half maximum (FWHM), crystallite size (*D*), and degree of crystallinity (%), were obtained using Diffrac.Eva software. The crystallite size (*D*) from XRD analysis was calculated by the Scherrer equation, which is eqn (1) below:1
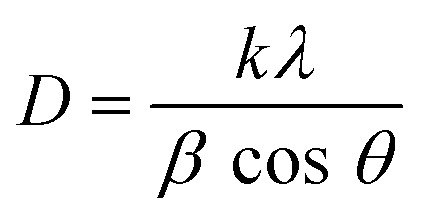
where *λ* is the wavelength of X-ray, *k* is the form factor or Scherrer constant, *θ* is the Bragg angle and *β* is the full width at half maximum (FWHM).

The modified Scherrer equation used to calculate the crystallite size is presented in eqn (2).2
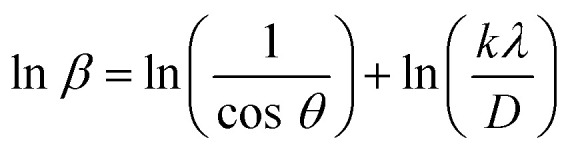


The Williamson–Hall (W–H) method was applied to determine both crystallite size and strain, as shown in eqn (3).3
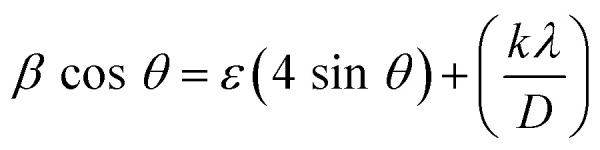


From the Williamson–Hall (W–H) plot, the crystallite size and strain were determined from the intercept and gradient, respectively.

The strain was calculated using the Scherrer eqn (4):4
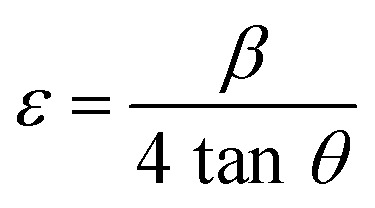


The type of strain was determined using eqn (5):5
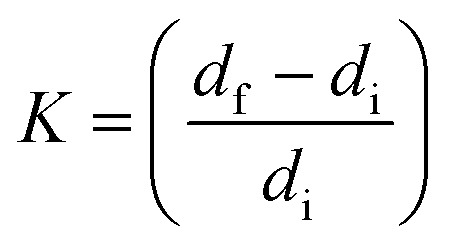
where *K* is the compressive or tensile strain, *d*_i_ is the interplanar spacing of undoped material, and *d*_f_ is the interplanar spacing of doped material. A positive *K* value indicates tensile strain (*d*_f_ > *d*_i_), whereas a negative *K* value indicates compressive strain (*d*_f_ < *d*_i_).

The dislocation density of the powder samples was calculated using the following eqn (6):6
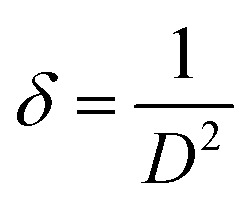


Thermo Scientific Raman spectroscopy analysis was conducted to study the structural and vibrational modes of the material. A DXR 2xi Raman imaging microscope equipped with 532 nm Ar ion laser was used. Spectra were recorded over the range of 50 cm^−1^ to 3400 cm^−1^ with a data spacing of 2 cm^−1^. The laser was focused directly on the surface of each powder sample, and measurements were taken from three different spots to ensure reproducibility. The integrated intensity ratio of disordered carbon (D band) and graphitic carbon (G band) was calculated using eqn (7):

7 Integrated intensity ratio = *I*_D_/*I*_G_

where, *I*_D_ is the intensity of the D band, and *I*_G_ is the intensity of the G band.

Brunauer–Emmett–Teller (BET) surface areas and pore size distribution were measured using a Micromeritics Tristar II plus instrument at 77 K. Prior to analysis, the samples were degassed at 200 °C for 3 hours. Dynamic light scattering (DLS) measurements were carried out using a Malvern (zeta sizer nano ZS) instrument for the suspension samples in ethanol. Particle size, zeta potential, mobility, and conductivity were determined from the DLS measurements. Field-emission scanning electron microscopy (FESEM) (MERLIN Compact, ZEISS, Germany) coupled with energy-dispersive X-ray (EDX) spectroscopy was used to examine the morphology and elemental distribution of the samples. FESEM images were obtained at an operating voltage of 3 keV with a magnification of 50k×, while EDX analysis was performed at 15 keV and a magnification of 5k×.

## 3. Results and discussion

### 3.1. FESEM with EDX/EDS analysis

**Fig. 2:**
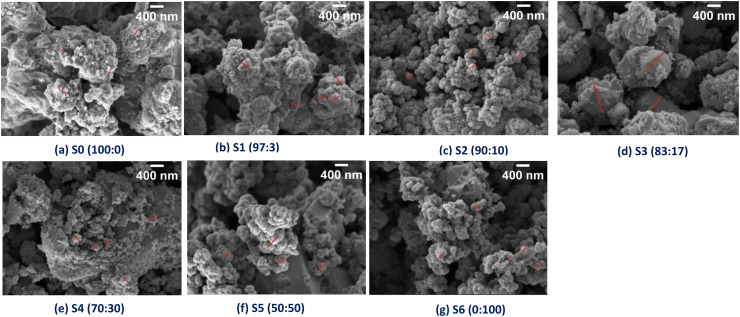
(a)–(g). The FESEM images of Mn-doped CZTS powder samples.

**Fig. 3:**
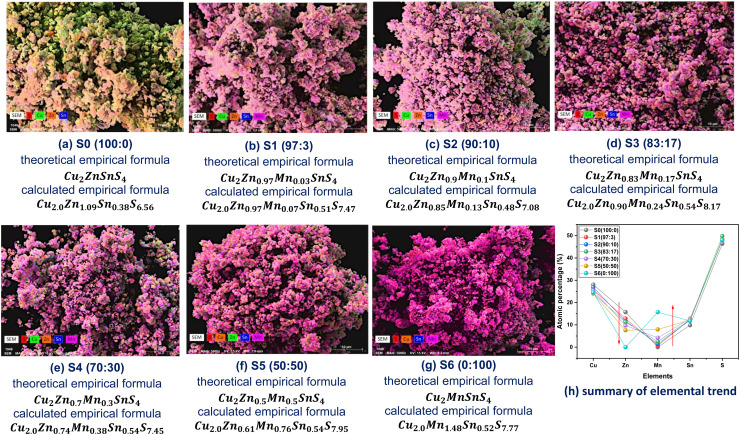
Mn-doped CZTS powder samples (a)–(g) EDS mapping & empirical formula, and (h) summary of elemental trend obtained.

FESEM images illustrate surface micrographs of Mn-doped CZTS powder samples. The surface micrographs of the Mn-doped CZTS samples using FESEM are presented in Fig. 2(a)–(g). The undoped CZTS samples exhibits larger agglomerates with irregular shapes and sizes. However, with Mn incorporation, the degree of agglomeration decreases noticeably. Sample S1 (97 : 3) displays plate-like and polyhedral shape particles with significant agglomeration, whereas S2 (90 : 10), S5 (50 : 50), and S6 (0 : 100) exhibit predominantly spherical shape particles. Furthermore, these samples show Craspedia flower-like spherical structure, which may enhance surface area and electrochemical activity.^41^ Among all samples, S3 (83 : 17) demonstrates a well-defined grain structure with high crystalline, consistent with XRD results discussed in Section 3.2. Samples S2 (90 : 10), S3 (83 : 17), and S4 (70 : 30) exhibit comparatively higher crystallinity, suggesting their suitability for various applications. The grain size from FESEM measurements is presented in Table 1. The grain size increases with Mn doping, reaching a maximum value for S3 (83 : 17), and then decreases at higher doping levels. This trend is consistent with particle size results obtained from DLS analysis. EDX/EDS analysis was conducted to confirm the elemental composition of the samples. The presence of Cu, Zn, Mn, Sn, and S was verified through EDS mapping, as shown in Fig. 3(a)–(g). The manganese percentage increases progressively, while the zinc content decreases from S0 (100 : 0) to S6 (0 : 100), as shown in Fig. 3(h), indicating successful Mn incorporation into the lattice. The empirical formula calculated for all samples are presented in Fig. 3(a)–(g). The calculated empirical formula agrees well with the theoretical form, confirming accurate compositional control and effective Mn substitution with the CZTS structure. Additionally, EDX/EDS mapping reveals a homogeneous spatial distribution of Mn throughout the CZTS matrix, with no observable phase segregation or clustering. The progressive increase in Mn mapping intensity with increasing doping concentration further confirms the controlled and uniform incorporation of Mn into the host lattice.

**Table 1:** Crystallographic information of Mn-doped CZTS at various (Zn : Mn) percentage ratios obtained from XRD analysis

Sample (Zn : Mn) in % ratio	FWHM, *β* (°)	Lattice, *a* (Å)	Lattice, *c* (Å)	Volume, *V* (Å^3^)	Interplanar spacing, *d*_112_ (nm)	Interplanar spacing, *d*_220_ (nm)	Strain, *ε* ×10^−3^ (rad)	Dislocation density, *δ* × 10^−3^ (nm)^−2^	Crystallite size, *D* (nm)	Degree of crystallinity (%)	Grain size from FESEM analysis (nm)
S0 (100 : 0)	0.3305	5.4248	10.8673	319.8078	0.3134	0.1918	5.69	1.67	24.52	72.06	106
S1 (97 : 3)	0.2896	5.4310	10.8875	321.1351	0.3138	0.1920	4.99	1.28	27.99	64.93	229
S2 (90 : 10)	0.1929	5.4329	10.8862	321.3215	0.3139	0.1921	3.32	0.57	42.01	53.46	170
S3 (83 : 17)	0.2392	5.4379	10.9005	322.3360	0.3142	0.1923	4.13	0.87	33.88	61.36	912
S4 (70 : 30)	0.2189	5.4403	10.8940	322.4282	0.3142	0.1923	3.76	0.72	37.14	57.97	471
S5 (50 : 50)	0.3798	5.4485	10.9110	323.9056	0.3147	0.1926	6.56	2.20	21.34	59.47	280
S6 (0 : 100)	0.4550	5.4997	11.1165	336.2374	0.3186	0.1944	7.97	3.16	17.79	56.05	273

### 3.2. XRD analysis

**Fig. 4:**
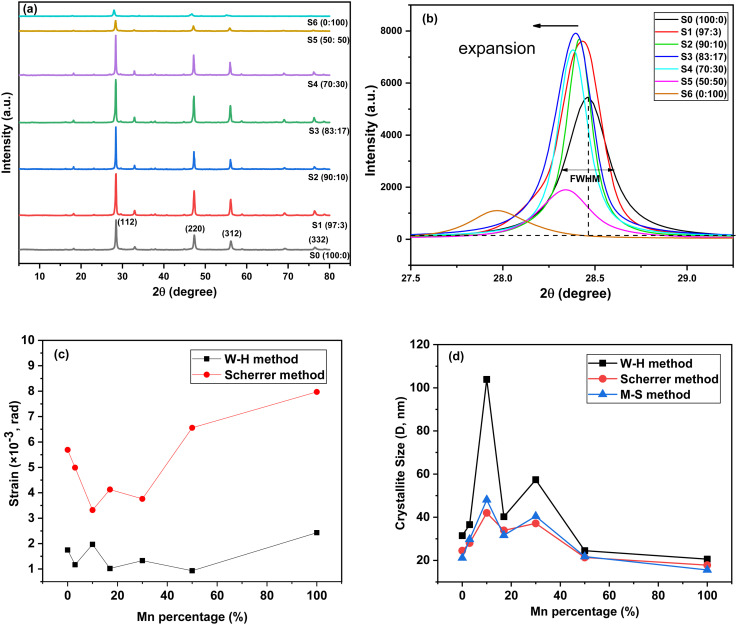
(a) The X-ray diffractogram of Mn-doped CZTS at various (Zn : Mn) percentage ratios, (b) enlargement XRD peaks of (112) planes, and various approximation method used to extrapolate FWHM to (c) lattice strain plot, and (d) crystallite size plot, for Mn-doped CZTS samples.

Fig. 4(a) presents the XRD diffractogram of Mn-doped CZTS powder samples at various Mn concentrations, while Fig. 4(b) shows an enlarged view of the (112) XRD peaks. The undoped sample S0 (100 : 0), exhibits characteristic diffraction peaks at 2*θ* = 28.5°, 47.3°, 56.2°, and 76.4°, corresponding to the (112), (220), (312), and (332) planes, respectively. These results are consistent with JCPDS 26-0575 and previously reported findings,^14^ confirming the tetragonal structure of the synthesised samples. Crystallographic parameters, including interplanar spacing (*d*), lattice position (*a*, *c*), volume (*V*), crystallite size (*D*) (refer eqn (1)), strain (*ε*) (refer eqn (4)), dislocation density (*δ*) (refer eqn (6)), full width at half maximum (FWHM), and degree of crystallinity (%), were extracted from the XRD diffractogram and summarised in Table 1.

Upon Mn incorporation, the peak of FWHM of (112) plane gradually shifts toward a lower diffraction angle, shown in Fig. 4(b). This systematic shift confirms the successful incorporation of the Mn into the CZTS lattice rather than the formation of secondary phases. The substitution of larger Mn^2+^ ions (0.80 Å) for smaller Zn^2+^ ions (0.74 Å) induce lattice distortion, increases the interplanar spacing (*d*), modifies the Bragg diffraction conditions, and results in the observed peak shift.^24,25,42^

The lattice expansion is first evidenced by the increase in interplanar spacing (*d*), confirmed by *d*_112_ and *d*_220_ planes, as presented in Table 1 and Fig. S1(a). Correspondingly, the lattice parameters ‘*a*’ and ‘*c*’ increase with increasing Mn content, as illustrated in Fig. S1(b). This expansion leads to an increase in unit cell expansion, as shown in Fig. S1(c). The calculated lattice parameters are in good agreement with reported reference values (*a* = 5.427 Å, and *c* = 10.848 Å).^14,43^ Fig. S1(d) illustrates the variation in strain, degree of crystallinity, and crystallite size with Mn concentration. At a low Mn doping level, the strain decreases, suggesting effective substitution of Mn ions into the lattice and a reduction in defect concentrations. In contrast, higher Mn concentrations result in increased strain due to local lattice distortion and defects formation.^44^ A similar trend is observed for dislocation density. The difference in ionic radii between Mn^2+^ and Zn^2+^ introduces structural distortions, and excessive Mn incorporation may generate interstitial defects that distort the surrounding lattice, thereby increasing the dislocation density.^45^

Fig. 4(c) illustrates lattice strain of Mn-doped CZTS at various loading, calculated using the Scherrer and Williamson–Hall (W–H) methods. The strain and FWHM exhibit a similar trend: a decrease in FWHM corresponds to a reduction in structural defects, and *vice versa*. Further, strain and crystallite size show an inverse correlation; as strain increases, crystallite size decreases, whereas reduced strain promotes crystallite growth. The crystallite size increases with Mn content up to sample S2 (90 : 10) and then decreases at higher doping levels. At the low Mn concentration, Mn ions substitute into existing vacancy defect sites, reducing defect density, as confirmed by the decrease FWHM and dislocation density as shown in Table 1. At sample S2 (90 : 10), most defect sites appear to be occupied by Mn ions, leading to reduced strain and enhanced grain coarsening and growth, as discussed in the FESEM section. This results in an increase in crystalline size. In contrast, at higher Mn concentration, excessive incorporation of larger Mn ions induces interstitial defects and lattice distortions due to ionic size mismatch. The resulting strain restricts grain boundary movement, thereby limiting grain coarsening and reducing crystallite size.^42^ The nature of the strain (compressive or tensile) was calculated using eqn (5) and is illustrated in Table S1. All samples exhibit positive strain values, confirming the presence of tensile strain in the doped materials.^46,47^ Furthermore, tensile strain values increase linearly with Mn content, indicating systematic structural distortion induced by dopant substitution.^46^

Because both crystallite size and lattice strain contribute to XRD peak broadening, Modified Scherrer (M–S) and Williamson–Hall (W–H) analyses were performed to identify the crystallite size and strain, in addition to the conventional Scherrer analysis discussed above and illustrated in Table S1. The strain calculated using the Scherrer method decreases at low Mn concentrations and increases at higher doping percentages, whereas the strain derived from the W–H method fluctuates, as shown in Fig. 4(c). This difference arises because the W–H approach accounts for anisotropic lattice distortion and local structural variations, making it more sensitive to complex strain behaviour.^48^ Thus, the non-synchronous trends between the two methods reflect the defect-strain interplay induced by Mn incorporation. The crystallite size obtained from the W–H method is consistently larger than that calculated using the Scherrer method as illustrated in Fig. 4(d), as the W–H approach separates strain contributions from size broadening. Importantly, both methods display a consistent compositional trend: crystallite size increases up to 10% Mn (S2) and decreases at higher Mn concentrations. The Modified Scherrer method yields crystallite sizes comparable to the conventional Scherrer approach.^49^ For instance, S2 (90 : 10) exhibits crystallite sizes of 42.01 nm (Scherrer) and 48.08 nm (M–S), while S4 (70 : 30) shows 37.14 nm (Scherrer) and 40.45 nm (M–S). The close agreement among these two methods confirms that the observed size variation is intrinsic and not significantly influenced by instrumental broadening. Overall, the W–H and M–S analyses corroborate the structural evolution identified by the Scherrer method. Fig. 4(b) shows that the intensity of the (112) peak increases with Mn concentration up to S3 (83 : 17) and then decreases, indicating enhanced crystallinity due to improved phase formation at moderate doping levels.^30^ However, the overall degree of crystallinity decreases after Mn incorporation compared to the undoped sample, suggesting increased lattice imperfections and chemical disorder. Among the Mn-doped samples, S1 (97 : 3) exhibits the highest degree of crystallinity. A nonlinear correlation is observed between degree of crystallinity and lattice expansion. Samples S0 (100 : 0), S1 (97 : 3), and S2 (90 : 10) exhibit mixed kesterite and stannite phases, whereas higher Mn concentrations favour the stannite phase exclusively, as confirmed by Raman analysis in Section 3.3. The kesterite phase typically exhibit higher degree of crystallinity than the stannite phase, resulting in a nonlinear variation in crystallinity and lattice expansion.^47,50^

Although the crystallite size at 10% Mn concentration (S2) increases significantly (42.01 nm) with a concurrent reduction in strain and dislocation density, the degree of crystallinity decreases relative to the undoped sample. This apparent contradiction arises from the distinction between crystallite growth and long-range structural ordering. Moderate Mn incorporation enhances atomic diffusion and grain coalescence. However, at approximately 10% substitution, Mn^2+^ incorporation disturbs the intrinsic Cu–Zn ordering within the kesterite lattice. Such cation redistribution and local symmetry distortion reduce long-range periodicity, even though coherent diffracting domains become larger. Therefore, while microstructural parameters (crystallite size, strain, dislocation density) indicate improved grain growth, the reduction in the degree of crystallinity reflects increased chemical disorder within the lattice.^51,52^

### 3.3. Raman analysis

**Fig. 5:**
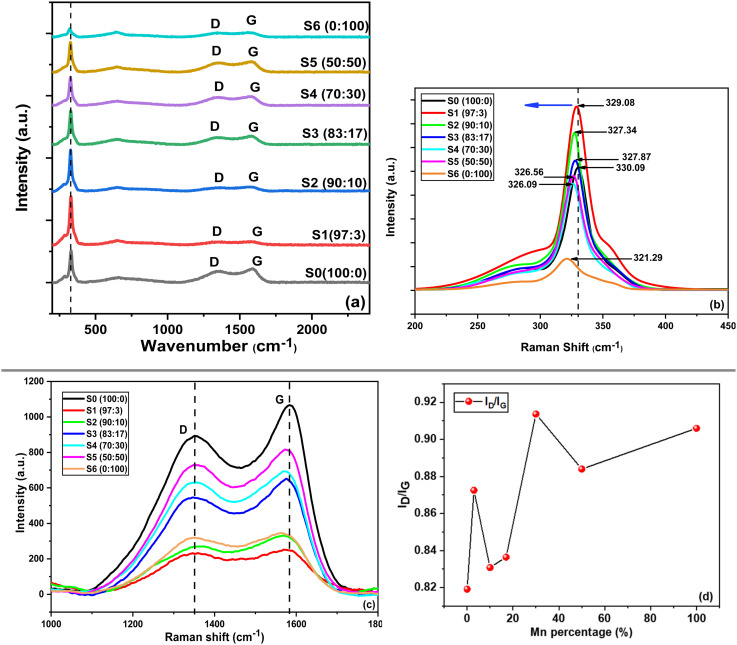
Raman spectra of Mn-doped CZTS at various (Zn : Mn) percentage ratio of (a) wide range between 200 and 2400 cm^−1^, (b) enlargement spectra between 200 and 450 cm^−1^, (c) enlargement spectra between 1000 and 1800 cm^−1^ for disordered carbon (D band) and graphitic carbon (G band) residue, and (d) *I*_D_/*I*_G_ plot *versus* Mn percentage loading.

Raman analysis for Mn-doped CZTS powder samples was carried out to identify the structural phases, as shown in Fig. 5(a). The most intense peak is recorded at 330 cm^−1^ for undoped CZTS and in the range of 321–329 cm^−1^ for Mn-doped CZTS, corresponding to the stannite phase of CZTS. These results are in excellent agreement with previous studies.^43,53^ Khare *et al.*^54^ simulated different Raman frequencies for CZTS and reported that the peak at 328–333 cm^−1^ is attributed to the A_1_ mode of the stannite phase (334.08 cm^−1^). A small shoulder peak is observed at 285 cm^−1^, which corresponds to the A mode of the kesterite phase (*P*4̄2*c* polymorphs) at 284.30 cm^−1^.^55^ This small shoulder peak is present in samples S0 (100 : 0), S1 (97 : 3), and S2 (90 : 10), but disappears at higher Mn concentrations. These observations indicate that Mn-doped samples are predominantly composed of the stannite phase, while the kesterite phase exists as a minor secondary phase in the undoped CZTS composition and low Mn-doped samples. For this study, the Raman spectra were obtained using Lorentzian deconvolution to obtain accurate peak positions and intensities. As shown in the enlarged view in Fig. 5(b), the major Raman peaks shift toward lower wavenumber with increasing Mn concentration. This “red shift” confirms the successful Mn substitution into the lattice. Moreover, the peak intensities increase after Mn doping, suggesting modifications in the electronic or structural properties of the material.^13^

The Raman spectra also exhibit additional peaks in the ranges of 1340–1355 cm^−1^ and 1575–1590 cm^−1^, corresponding to disordered carbon (D band) and graphitic carbon (G band) shown in Fig. 5(c).^25^ These bands originate from residual carbonaceous species formed during the decomposition of acetate precursors. The G band arises from the in-plane stretching vibration between sp^2^ carbon atoms, whereas the D band is associated with structural defects, edge effects, and vacancies. The *I*_D_/*I*_G_ ratio as in eqn (7), indicates the degree of disorder or defect density.^56^ As illustrated in Fig. 5(d), the *I*_D_/*I*_G_ ratio increases with increasing Mn concentration, indicating a rise in defect density within the CZTS lattice. This trend suggests that the ionic size mismatch between Mn^2+^ and Zn^2+^ introduces additional lattice distortion and vacancy-related defects. The resulting structural disorder may alter the electronic structure and local bonding environment, thereby influencing the surface characteristics and charge transfer properties of the material.

### 3.4. BET analysis

**Fig. 6:**
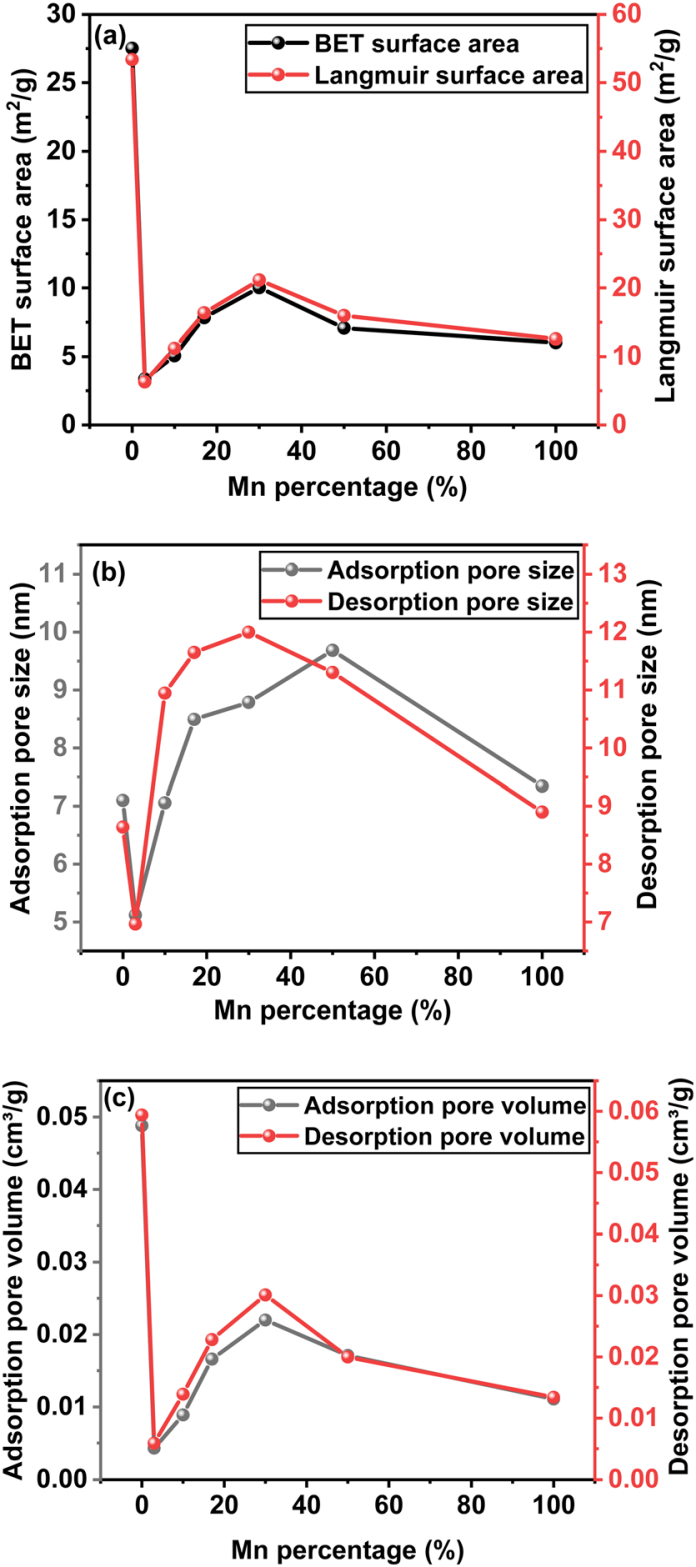
BET analysis of Mn-doped CZTS samples (a) surface area, (b) pore size, and (c) pore volume.

BET analysis for Mn-doped CZTS samples was carried out to evaluate the surface characteristics and porosity. The BET N_2_ adsorption–desorption isotherms of Mn-doped CZTS powders are presented in Fig. S2. The samples exhibit type IV profile with an H2-type hysteresis loop, confirming the mesoporous nature. At low relative pressures (*P*/*P*_0_ < 0.2), minimal N_2_ adsorption is observed, indicating limited microporosity. A gradual increase in adsorption occurs in the intermediate pressure range (0.2 < *P*/*P*_0_ < 0.8), consistent with the multilayer adsorption on mesopore surfaces. A sharp increase in the adsorbed volume at higher pressures (*P*/*P*_0_ > 0.8) is attributed to capillary condensation within the mesopores. The presence of an H2-type hysteresis loop, arising from the difference between adsorption and desorption branches, suggests the presence of ink-bottle type pores with narrow necks and wider pore bodies.^57^ The BET surface area and pore volume decrease due to doping, while pore size increases significantly, as shown in Fig. 6(a)–(c). The reduction in BET surface area is attributed to the increase in particle size after the doping, consistent with the DLS findings. However, among the doped samples, the BET surface area, pore volume, and pore size increases with Mn content up to S4 (70 : 30) and then decreases at higher doping levels, as summarised in Table S2. The maximum BET surface area (10.0245 m^2^ g^−1^), pore size (11.9997 nm), and pore volume (0.0220 cm^3^ g^−1^) are observed for sample S4 (70 : 30). In contrast, the lowest values – 3.3603 m^2^ g^−1^ (surface area), 6.9673 nm (pore size), and 0.0043 cm^3^ g^−1^ (pore volume) – are recorded for S1 (97 : 3). The increase in pore size following Mn incorporation is attributed to enhanced lattice strain. Increased strain restricts grain coarsening and growth, leading to smaller, irregularly shaped, and misaligned grains. This microstructural evolution creates intergranular gaps, looser interparticle packing, and void formation, thereby increasing the pore size.^33^

### 3.5. DLS analysis

**Fig. 7:**
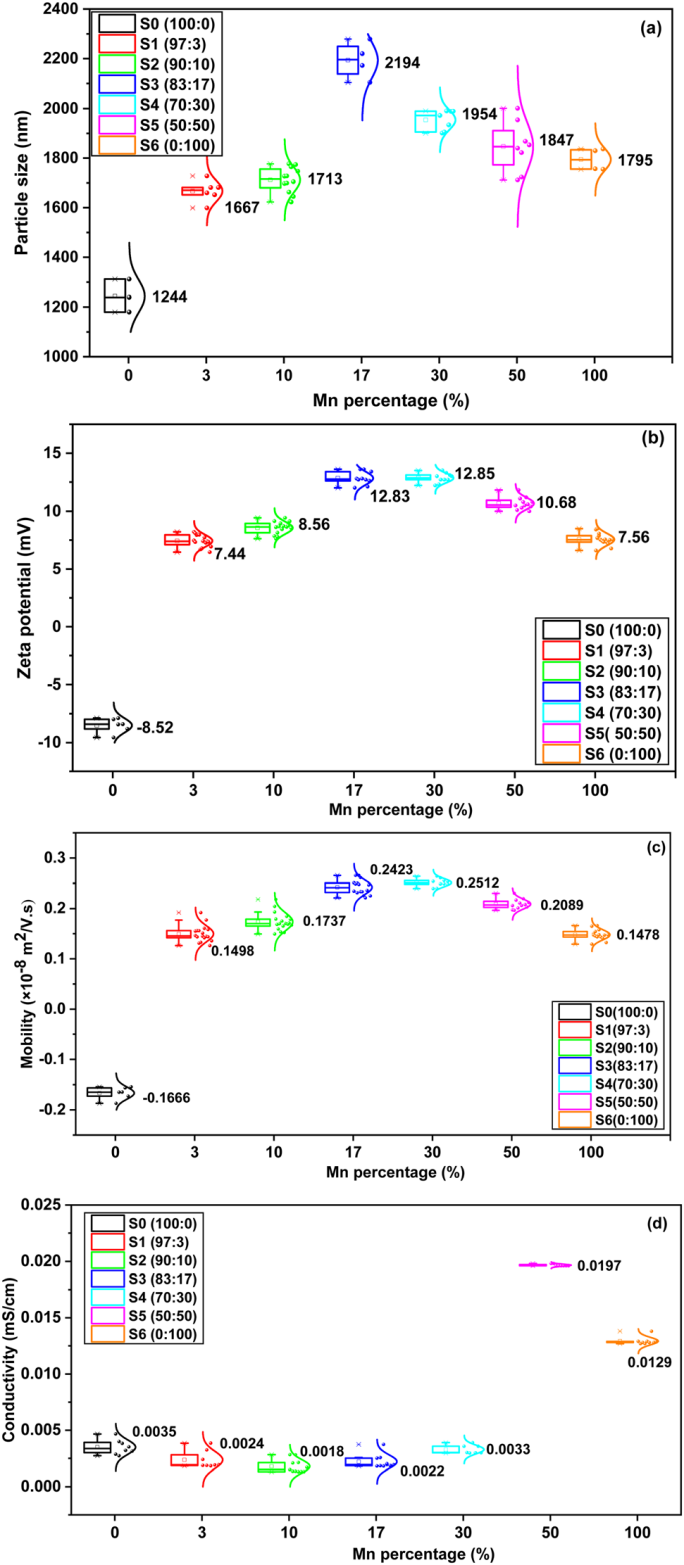
DLS information obtained for Mn doped CZTS at various percentage (a) particle size, (b) zeta potential, (c) mobility, and (d) conductivity.

Dynamic light scattering (DLS) measurements were carried out to determine the particle size, zeta potential, mobility, and conductivity of Mn-doped CZTS samples at various doping levels, as shown in Fig. 7. The particle size distributions are presented in Fig. S3. The polydispersity index (PDI) values range from 0.1 to 0.6, indicating a moderately polydisperse system with controlled particle size distribution without severe aggregation.^58,59^ While these values suggest reasonable uniformity in particle population, dopant homogeneity was further verified using compositional analyses, particularly Mn spatial distribution by EDX analysis rather than inferred solely from PDI values. As illustrated in Fig. 7(a), the particle size increases progressively up to S4 (70 : 30) and then decreases at higher Mn concentrations. This pattern is attributed to Mn incorporation into the lattice system, which promotes grain enlargement at moderate doping levels. Increased particle size, associated with larger grains, may improve crystallinity and reduce grain boundary resistance, thereby enhancing material stability.

The electrical charge distribution of the Mn-doped CZTS powders dispersed in ethanol is reflected by zeta potential as summarized in Fig. 7(b). The undoped CZTS particles show a negative zeta potential (−8.52 mV), consistent with sulphur-terminated surfaces and deprotonated surface groups that impart a net negative charge in aqueous suspension. Upon Mn incorporation, the zeta potential shifted to positive values (7–13 mV), indicating a significant change in the surface charge environment. During *in situ* doping, Mn^2+^ incorporation into the CZTS lattice may create local charge imbalances depending on the substitution site. Replacement of Zn^2+^ maintains charge neutrality; however, substitution at Cu^+^ or Sn^4+^ positions introduce local charge mismatch. This imbalance may be compensated by cation vacancy formation or partial oxidation of Mn^2+^ to higher oxidation states (Mn^3+^/Mn^4+^). In addition, the redox-active nature of Mn allows partial surface oxidation during high temperature treatment or post synthesis exposure. These mechanisms enrich the particle surface with positively charged species, contributing to the observed positive zeta potential. Thus, the transition from negative (undoped) to positive (Mn-doped) zeta potential is attributed to Mn-driven alterations of surface states through lattice substitution, charge compensation mechanisms, and surface oxidation, which together promote a cation-rich surface.^60,61^

Zeta potential and electrophoretic mobility demonstrate similar behaviour, both increasing with the manganese percentage up to S4 (70 : 30) and subsequently declining at higher doping levels. The increase in zeta potential indicates enhanced colloidal stability due to stronger electrostatic repulsion between particles. In colloid system, dispersions with zeta potential greater than 30 mV are generally considered electrostatically stable, whereas values below 10 mV indicate a tendency toward aggregation due to repulsive forces.^62^ In this study, the undoped CZTS exhibited a zeta potential of −8.52 mV, indicating weak colloidal stability. Mn-doped CZTS samples show between 7 to 13 mV, exhibiting moderate stability of the colloidal. The electrophoretic mobility increases with zeta potential as illustrated in Fig. 7(c). This increase in surface charge results in stronger electrostatic force under an electric field, leading to faster particle motion.

The conductivity values derived from DLS provides further insight into ionic environment of the dispersions as shown in Fig. 7(d). The undoped CZTS shows a conductivity of 0.0035 mS cm^−1^. In contrast, Mn-doped CZTS, such as S1 (97 : 3), S2 (90 : 10), S3 (83 : 17), and S4 (70 : 30), display slightly reduced conductivity compared to undoped CZTS. This behaviour suggests that reduced ion release and improved lattice stability following Mn substitution. At higher Mn doping concentrations, however, conductivity increases sharply, which may result from incomplete Mn incorporation into the lattice, improved surface ion dissolution, or the presence of residual precursor species. The higher ionic strength at elevated doping levels can compress the electrical double layer, thereby reducing the zeta potential of these samples.^62–64^ A correlation between conductivity and degree of crystallinity is observed at low Mn concentrations. However, at higher doping levels, conductivity increases despite a reduction in crystallinity. This apparent discrepancy is consistent with the literature reported by Çolak *et al.*^41^ that the introduction of Mn^2+^ into the CZTS lattice (forming Cu_2_MnSnS_4_-related structures) creates additional electronic states that enhance charge carrier dynamics, and as a result, improve electrical conductivity. This boosts the photocatalytic activity. Similarly, Lie *et al.*^27^ reported that Mn-doped CZTSSe exhibits a substantial increase in carrier concentration and hole mobility beyond 40% of Mn doping, leading to significantly enhanced conductivity. Although the conductivity trend does not follow the crystallinity at a higher percentage of Mn doping, this difference is expected as per the literature studied.

## 4. Conclusions

Mn-doped Cu_2_ZnSnS_4_ samples with different percentage ratios of manganese were successfully synthesised using sol–gel method. XRD and Raman analyses confirmed the formation of tetragonal stannite structure without detectable secondary phases. The systematic shift of XRD peak toward lower diffraction angle verified the successful incorporation of Mn into the CZTS lattice. Mn substitution significantly influenced the structural lattice parameters including crystallite size, degree of crystallinity, strain, and dislocation density. Sample S2 (90 : 10) exhibited the largest crystallite size along with reduced strain and dislocation density, indicating improved structural ordering. Sample S4 (70 : 30) showed a moderate crystallite size (37.14 nm), which is suitable for electrochemical energy storage applications. The highest degree of crystallinity (64.93%) was observed for S1 (97 : 3), which is advantageous for enhanced optical absorption, carrier mobility, and conductivity, making it suitable for photovoltaic and solar cell applications. In contrast, S3 (83 : 17), with a moderate crystallinity (61.36%), presents a balanced defect concentration that could benefit supercapacitors, supercapatteries, and rechargeable battery applications. Additionally, sample S3 (83 : 17) also showed the biggest particle size, which improves electron mobility by reducing carrier recombination and grain boundaries, and minimizing charge scattering. This characteristic also makes it beneficial electrochemical energy storage, thermoelectric, and sensor applications. BET analysis revealed type IV isotherms with H2 hysteresis loops, confirming the mesoporous nature of the samples. The highest specific surface area was obtained for S4 (70 : 30), while relatively larger pore sizes were observed for S3 (83 : 17), S4 (70 : 30), and S5 (50 : 50). The combination of high surface area and enlarged pore size enhances the availability of active sites and facilitates ion diffusion and charge–discharge kinetics, which are essential for electrochemical energy storage systems. DLS analysis showed that S3 (83 : 17), S4 (70 : 30), and S5 (50 : 50) possess improved electrostatic stability, as indicated by their zeta potential and mobility values, supporting better dispersion stability and homogenous thin-film formation. The highest electrophoretic mobility was recorded for S4 (70 : 30), suggesting enhanced charge transport characteristics beneficial for electrochemical, photovoltaic, and sensing applications. Electrical conductivity decreased for S1–S4 compositions, indicating improved lattice stability upon Mn incorporation, which may be advantageous for battery, photovoltaic, dielectric, and protective coating applications. Conversely, S5 (50 : 50) exhibited higher conductivity, implying reduced internal resistance and potential suitability for high-performance energy storage and energy harvesting devices. EDX analysis confirmed the successful Mn incorporation into the CZTS, while FESEM results supported the particle size trends analysis from XRD and DLS results. In summary, samples S3 (83 : 17), S4 (70 : 30), and S5 (50 : 50) demonstrates a balanced combination of structural, morphological, surface, and electric properties, making them promising candidates for further investigation in electrochemical energy storage, and multifunctional energy-related applications.

## Author contributions

Ahamed Razeek Najitha: writing – original draft, methodology, investigation, data curation and formal analysis, visualization; Siti Rudhziah Che Balian: supervision, writing – review & editing; Hassan Ahmoum: supervision, methodology, validation, writing – review & editing; Fatin Saiha Omar: supervision, validation, writing – review & editing; Puvaneswaran Chelvanathan: supervision, methodology, resources, validation, writing – review & editing; and Mohd Sukor Su'ait: supervision, conceptualization, methodology, resources, validation, writing – review & editing, project administration and funding acquisition.

## Conflicts of interest

The authors declare that there are no conflicts of interest.

## Supplementary Material

RA-016-D5RA09688J-s001

## Data Availability

The data supporting the findings of this study are not publicly available due to confidentiality considerations. However, the data is available from the corresponding author upon reasonable request. Supplementary information (SI) is available. See DOI: https://doi.org/10.1039/d5ra09688j.
