# Supplementary material for: Manganese (Mn) doping effects on the structure and surface characteristics of copper zinc tin sulphide (CZTS) transition metal sulphides synthesised via a sol–gel method
Source: RSC Adv. 2026 Mar 27;16(19):16993–7004. doi: 10.1039/d5ra09688j (PMC13025148; doi:10.1039/d5ra09688j)
Supplement: RA-016-D5RA09688J-s001 [file RA-016-D5RA09688J-s001.pdf]

Supplementary Information (SI) for RSC Advances.
This journal is © The Royal Society of Chemistry 2026

SUPPLEMENTARY INFORMATION

Figure S1 - XRD analysis

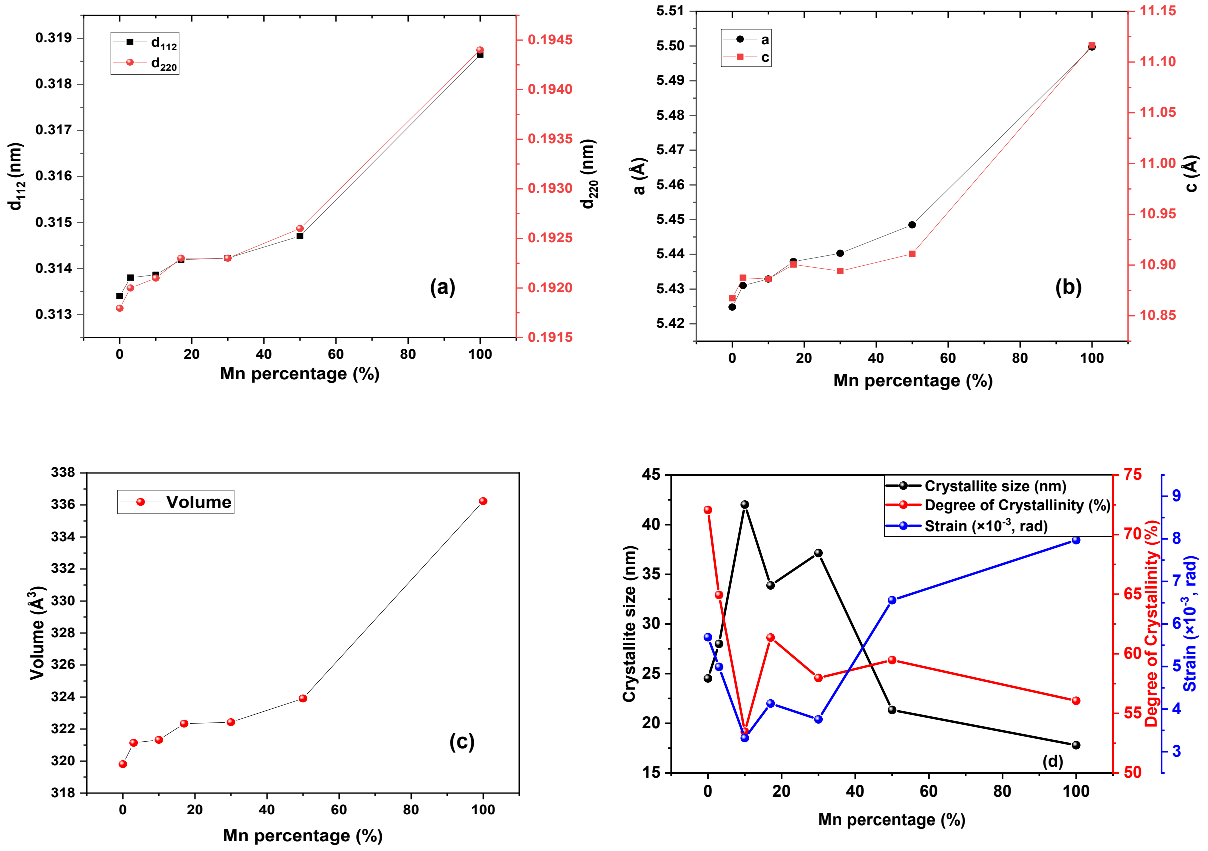

Figure S1 (a) interplanar spacing for various Mn percentages, **(b)** Lattice parameters ‘a’ and ‘c’ for Mn doped CZTS samples, and **(c)** volume for Mn doped CZTS samples **(d)** crystallite size, degree of crystallinity, and strain for Mn doped CZTS samples.

Table S1. The crystallite size and strain for Williamson-hall method, Scherrer method and modified Scherrer method and calculated strain values to identify nature of strain

Sample (Zn:Mn) percentage ratio	Williamson–Hall method		Scherrer Method		Modified Scherrer method	Nature of strain calculation		
	Crystallite Size (*D*) (nm)	Strain, $\varepsilon\times10^{-3}$ (rad)	Crystallite Size (*D*) (nm)	Strain, $\varepsilon\times10^{-3}$ (rad)	Crystallite Size (*D*) (nm)	$K\ (d_{112})$	$K\ (d_{220})$	Avg. strain (*K*)
**S0 (100:0)**	31.45	1.75	24.52	5.69	21.10	0	0	0.0000
**S1 (97:3)**	36.56	1.17	27.99	4.99	29.72	0.0013	0.0010	0.0012
**S2 (90:10)**	103.87	1.97	42.01	3.32	48.08	0.0015	0.0016	0.0015
**S3 (83:17)**	40.21	1.02	33.88	4.13	31.62	0.0025	0.0026	0.0026
**S4 (70:30)**	57.37	1.33	37.14	3.76	40.45	0.0026	0.0026	0.0026
**S5 (50:50)**	24.48	0.93	21.34	6.56	21.78	0.0042	0.0042	0.0042
**S6 (0:100)**	20.59	2.43	17.79	7.97	15.58	0.0167	0.0136	0.0151

Figure S2 - BET analysis

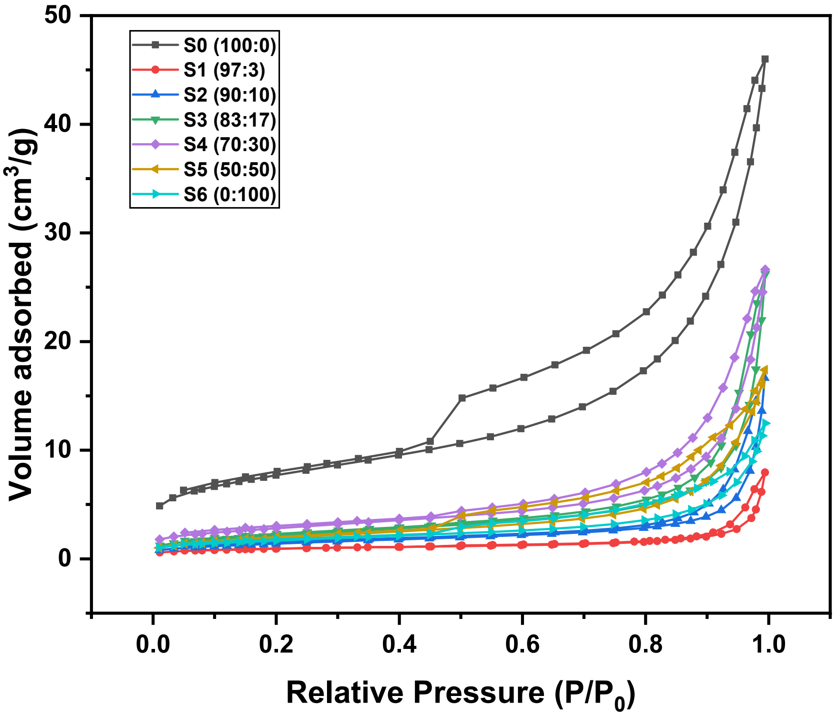

Figure S2. BET-N_2 adsorption-desorption isotherm

Table S2: Surface area and pore size for all anode samples.

**Sample (Zn:Mn) percentage ratio**	**BET Surface area, m^2/g**	**Langmuir Surface area, m^2/g**	**Adsorption pore size, nm**	**Desorption pore size, nm**	**adsorption pore volume (cm^3/g)**	**desorption pore volume (cm^3/g)**
**S0 (100:0)**	27.5130	53.3894	7.1012	8.6385	0.0488	0.0594
**S1 (97:3)**	3.3603	6.3028	5.1224	6.9673	0.0043	0.0059
**S2 (90:10)**	5.0669	11.1901	7.0569	10.9467	0.0089	0.0139
**S3 (83:17)**	7.8286	16.3453	8.4935	11.6504	0.0166	0.0228
**S4 (70:30)**	10.0245	21.1536	8.7884	11.9997	0.0220	0.0301
**S5 (50:50)**	7.0641	15.9444	9.6853	11.3034	0.0171	0.01996
**S6 (0:100)**	6.0252	12.5485	7.3430	8.8983	0.0111	0.0134

Figure S3. DLS analysis

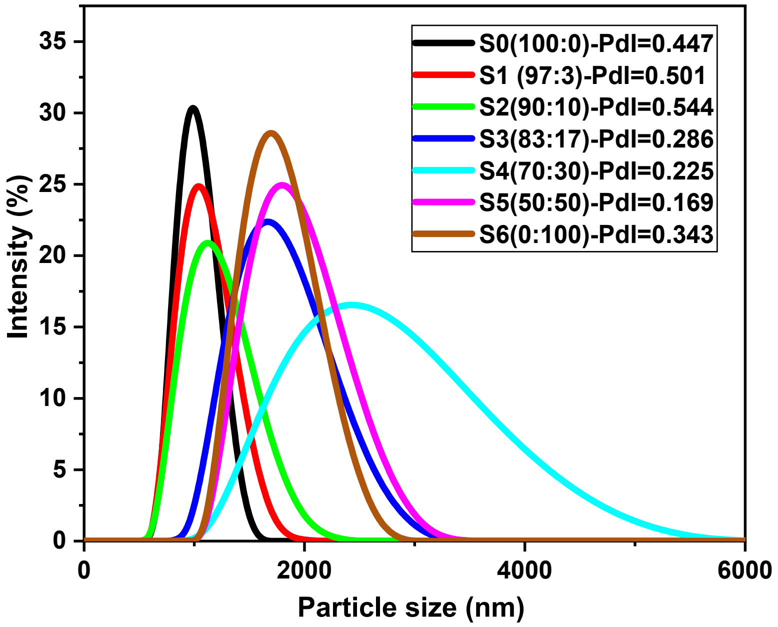

Figure S3. particle size distribution from DLS measurements
